# Supplementary material for: Engineering yeast for the production of breviscapine by genomic analysis and synthetic biology approaches
Source: Nat Commun. 2018 Jan 31;9:448. doi: 10.1038/s41467-018-02883-z (PMC5792594; doi:10.1038/s41467-018-02883-z)
Supplement: Supplementary file 1 — Supplementary Information [file 41467_2018_2883_MOESM1_ESM.docx]

**Engineering yeast for the production of breviscapine by genomic analysis and synthetic biology approaches**

Liu *et al.*

**
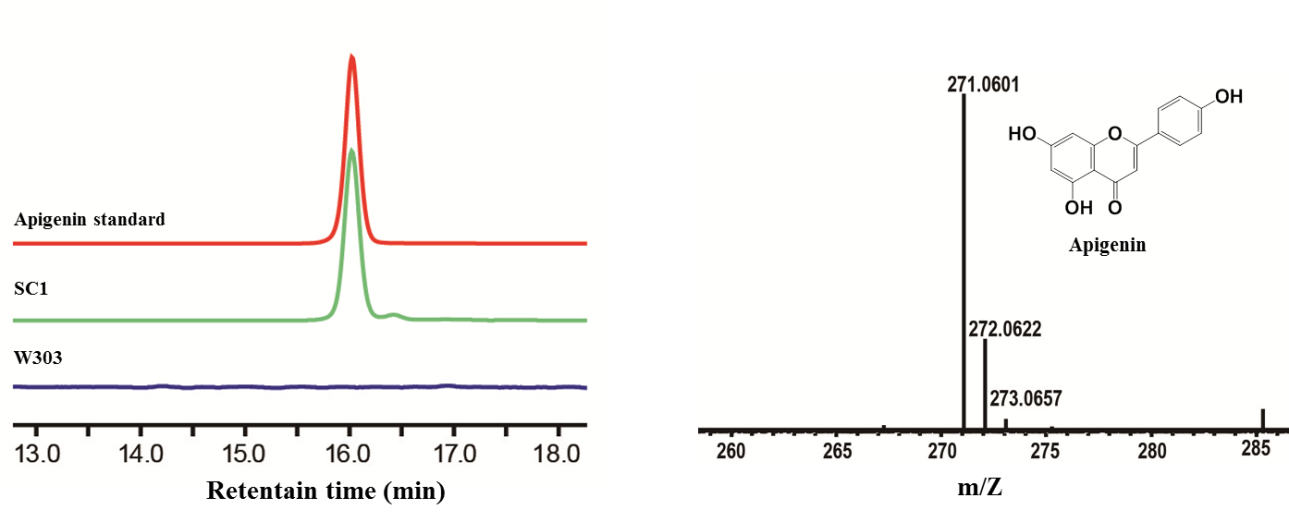
**

**Supplementary Figure 1 | HPLC-MS analysis to determine the production of apigenin.** (a) HPLC analysis of apigenin standard, fermentation products of strain SC1 and wild type W303-1B. (b) MS analysis of apigenin in the fermented products of SC1.

**
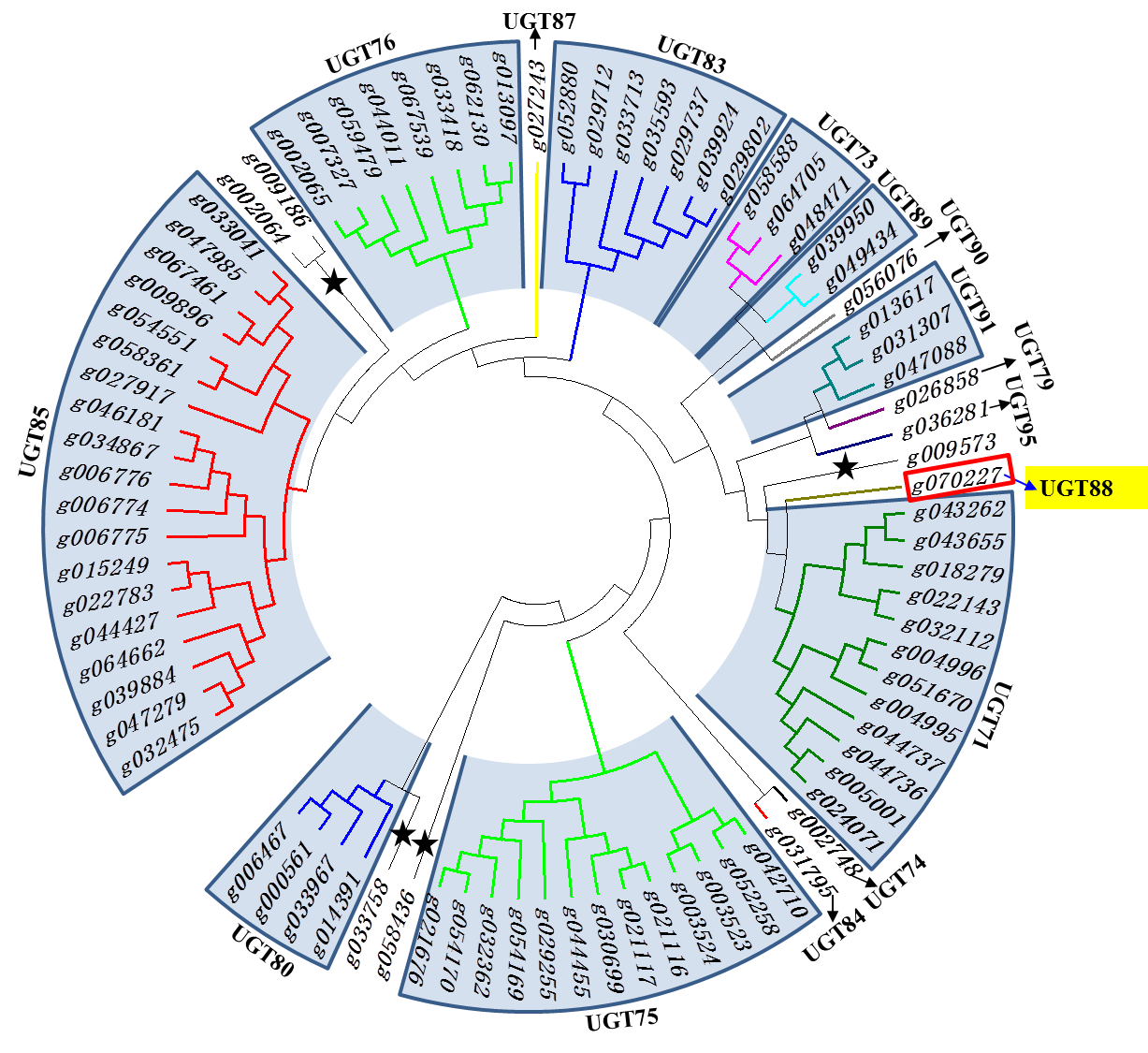
**

**Supplementary Figure 2 |** **The phylogenetic tree of 83** **UDPGT genes from *E. breviscapus* genome.** All UDPGT genes were identified by the UDPGT hmm model PF00201 (E-value < 1E^-10^) from Pfam and with protein length from 400 to 650. Among of them, 78 UDPGT genes can be assigned into 15 gene families based on the previous study^1^, only one gene belongs to UGT88 family. The maximum-likelihood tree was constructed by MEGA^2^.

**
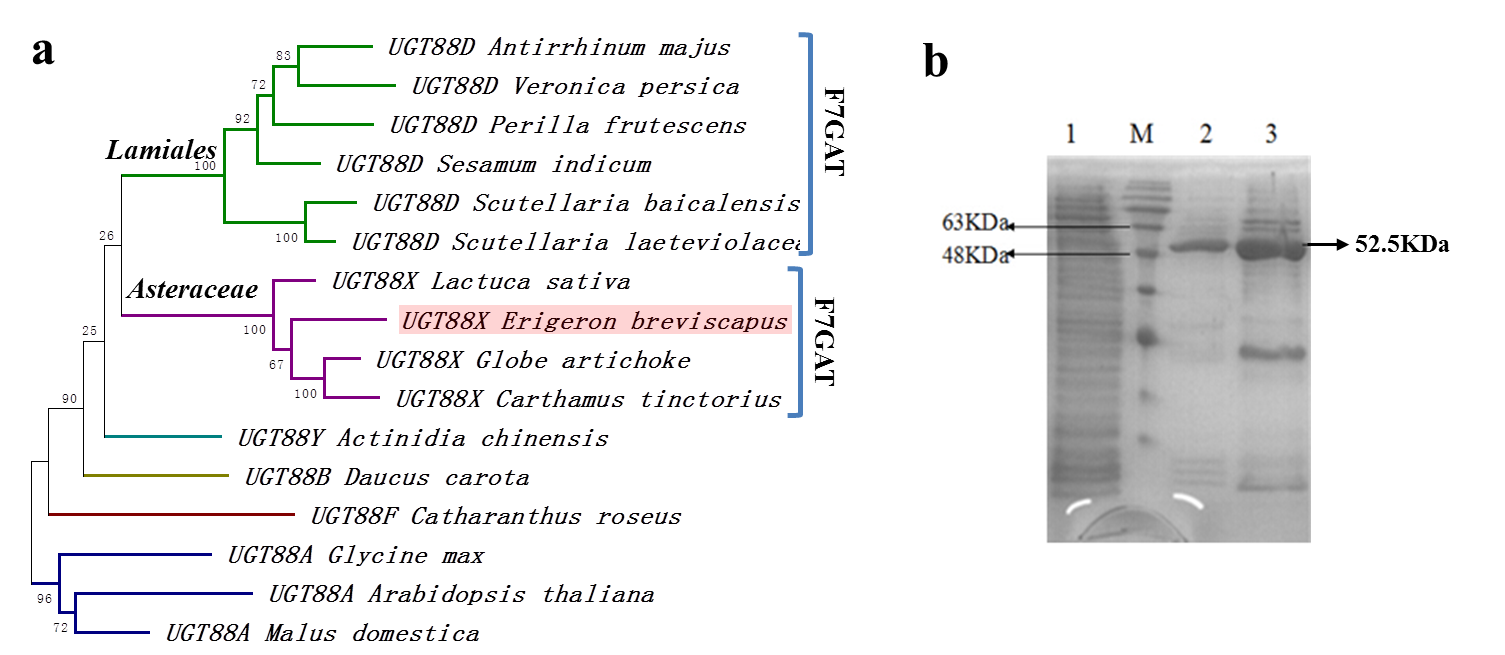
**

**
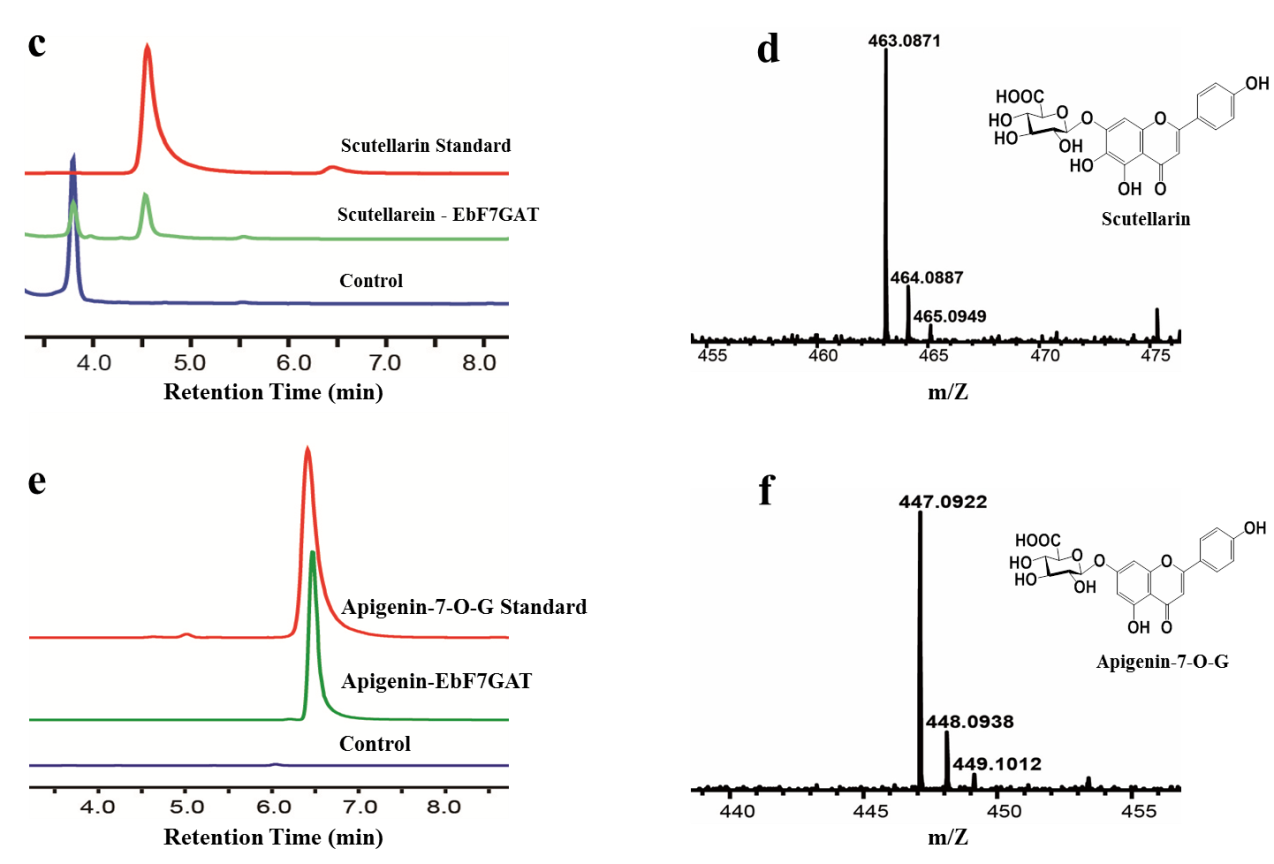
**

**Supplementary Figure 3 |** **Functional verification of EbF7GAT enzyme*.*** (**a**) The maximum-likelihood tree of UGT88 family. Most reported F7GATs belong to UGT88D subfamily^3, 4^ (green branch). In our study, the identified F7GAT in *E. breviscapus* belongs to a new subfamily UGT88X (purple branch). The protein sequences were shown in Supplementary Data 4. The tree was constructed by MEGA^2^. (**b**) SDS-PAGE analysis of the purified EbF7GAT. (**c**) HPLC analysis of the EbF7GAT enzyme activity with scutellarein as substrate. (**d**) MS analysis of scutellarin in enzyme reaction system with scutellarein as substrate. (**e**) HPLC analysis of the EbF7GAT enzyme activity with apigenin as substrate. (**f**) MS analysis of apigenin-7-O-glucuronide in enzyme reaction system with apigenin as substrate.

**
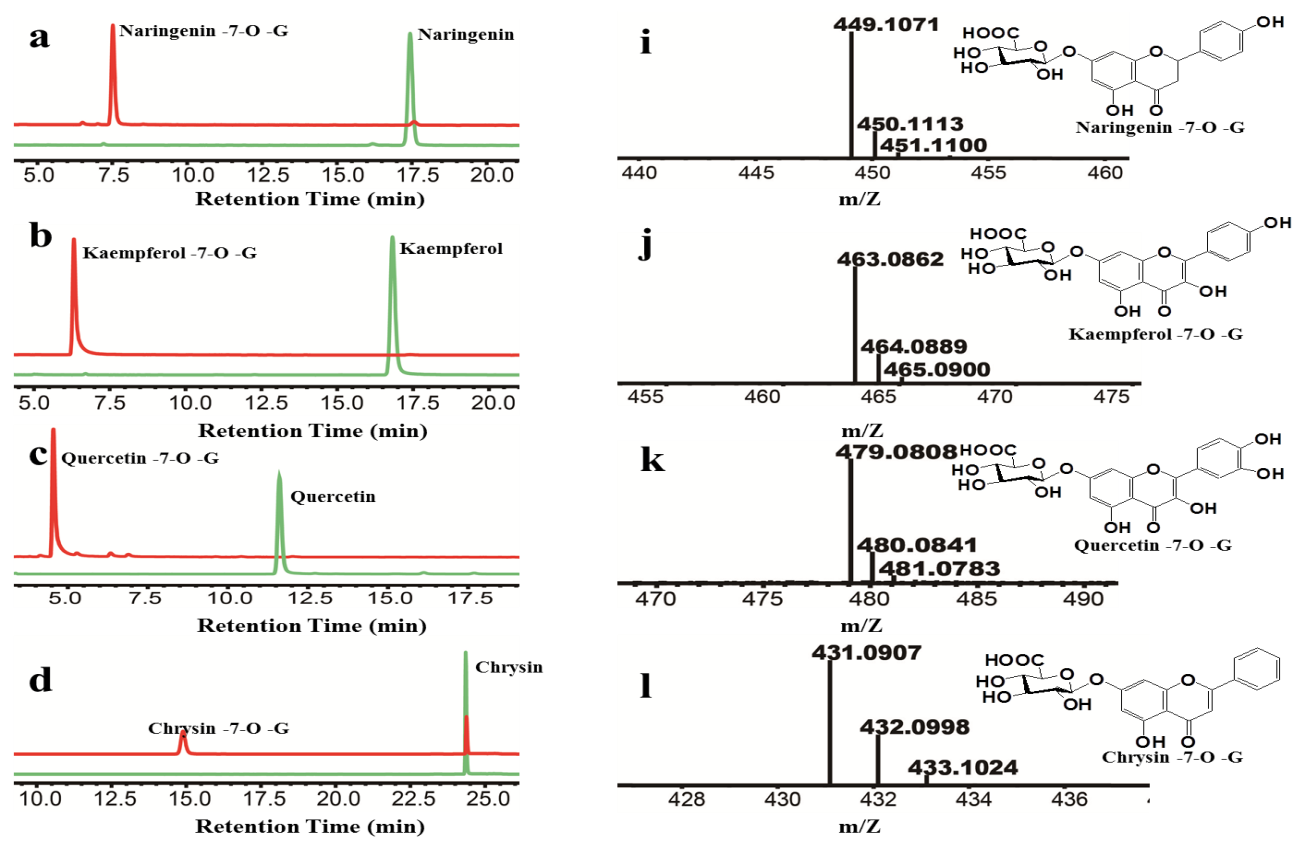
**

**
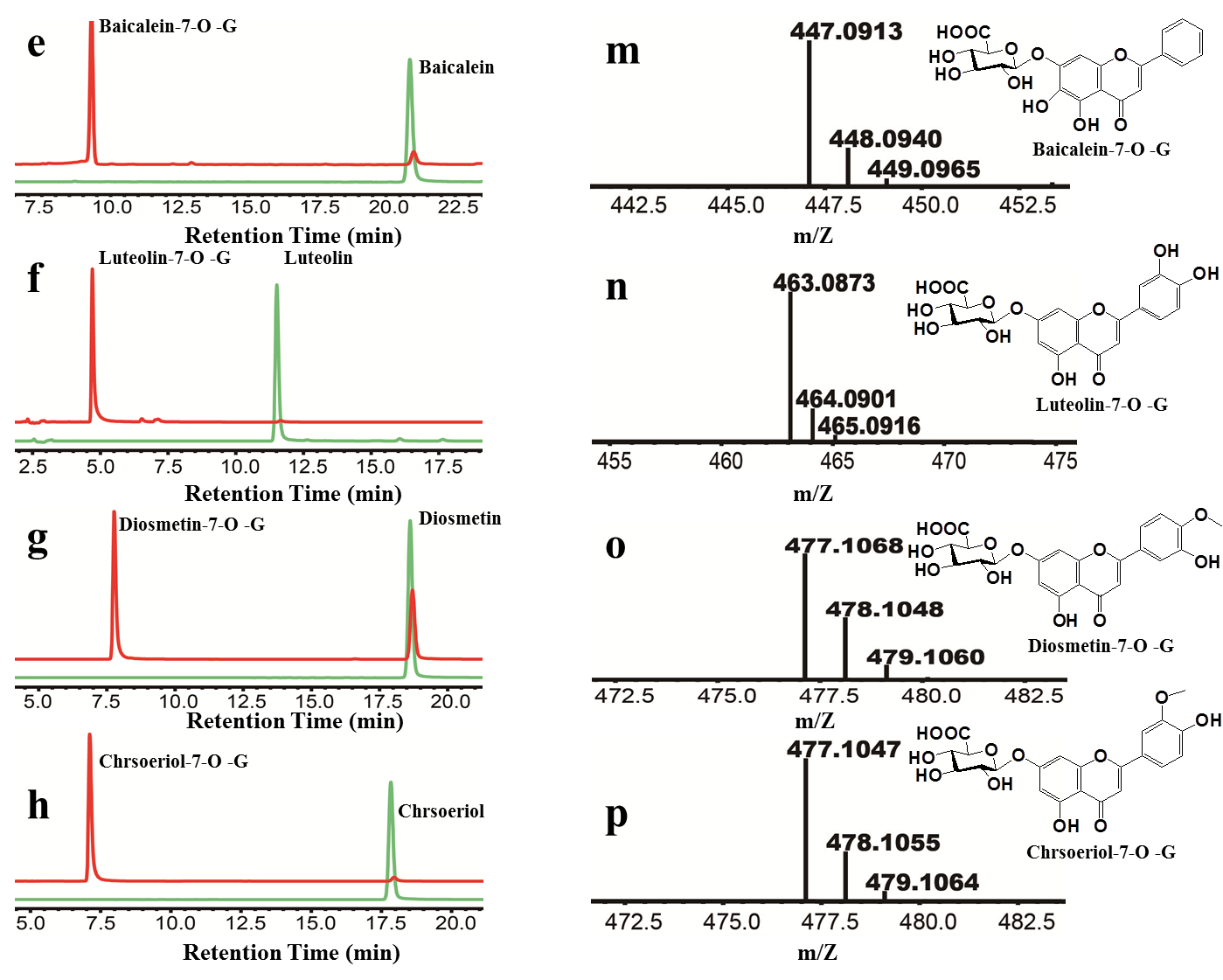
Supplementary Figure 4 | Screening of substrates spectrum for EbF7GAT enzyme*.*** (**a-e**) HPLC analysis of the EbF7GAT enzyme reaction products with naringenin, kaempferol, quercetin, chrysin, baicalein, luteolin, diosmetin, chrysoeriol as substrates *in vitro*. (**f-p**) MS analysis of the corresponding flavonoid-7-O-glucuronide products with naringenin, kaempferol, quercetin, chrysin, baicalein, luteolin, diosmetin, chrysoeriol as substrates.

**
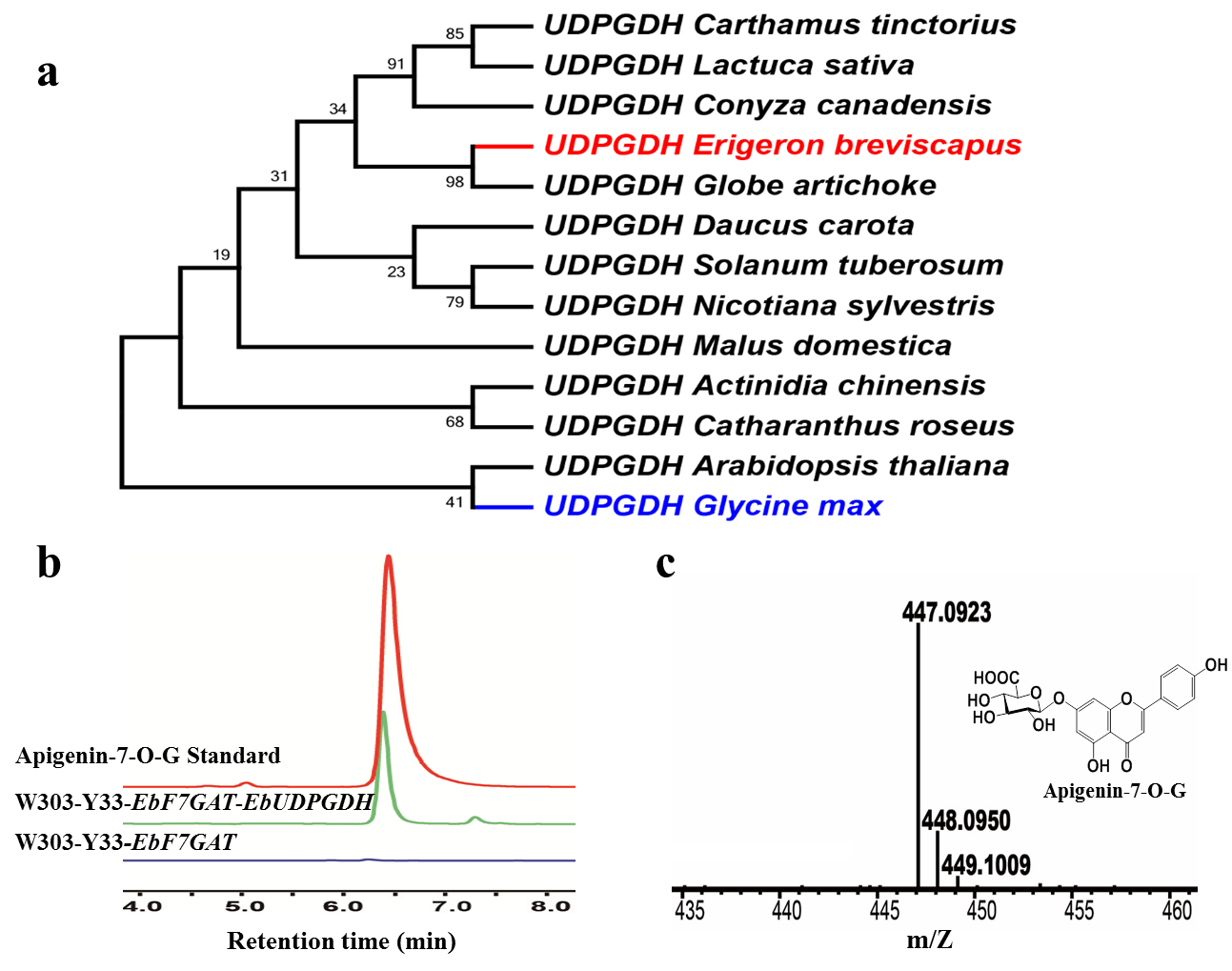
**

**Supplementary Figure 5 | Functional verification of EbUDPGDH enzyme*.*** (**a**) Phylogenetic relationship of *UDPGDH* genes. The *UDPGDH* gene in *E. breviscapus* (Ebre_g026582, red color) was predicted based on the orthologous gene in *Glycine max* (blue color). The *UDPGDH* gene had a high amino-acid identity between *E. breviscapus* and *Glycine max* (91%). The maximum-likelihood tree was constructed by MEGA^2^. (**b**) HPLC analysis of apigenin-7-O-glucuronide in the fermented products of strain W303-Y33-*EbF7GAT*-*EbUDPGDH* by feeding apigenin as substrate. W303-Y33-*EbF7GAT* was used as negative control. (**c**) MS analysis of apigenin-7-O-glucuronide in the fermented product of W303-Y33-*EbF7GAT*-*EbUDPGDH*.


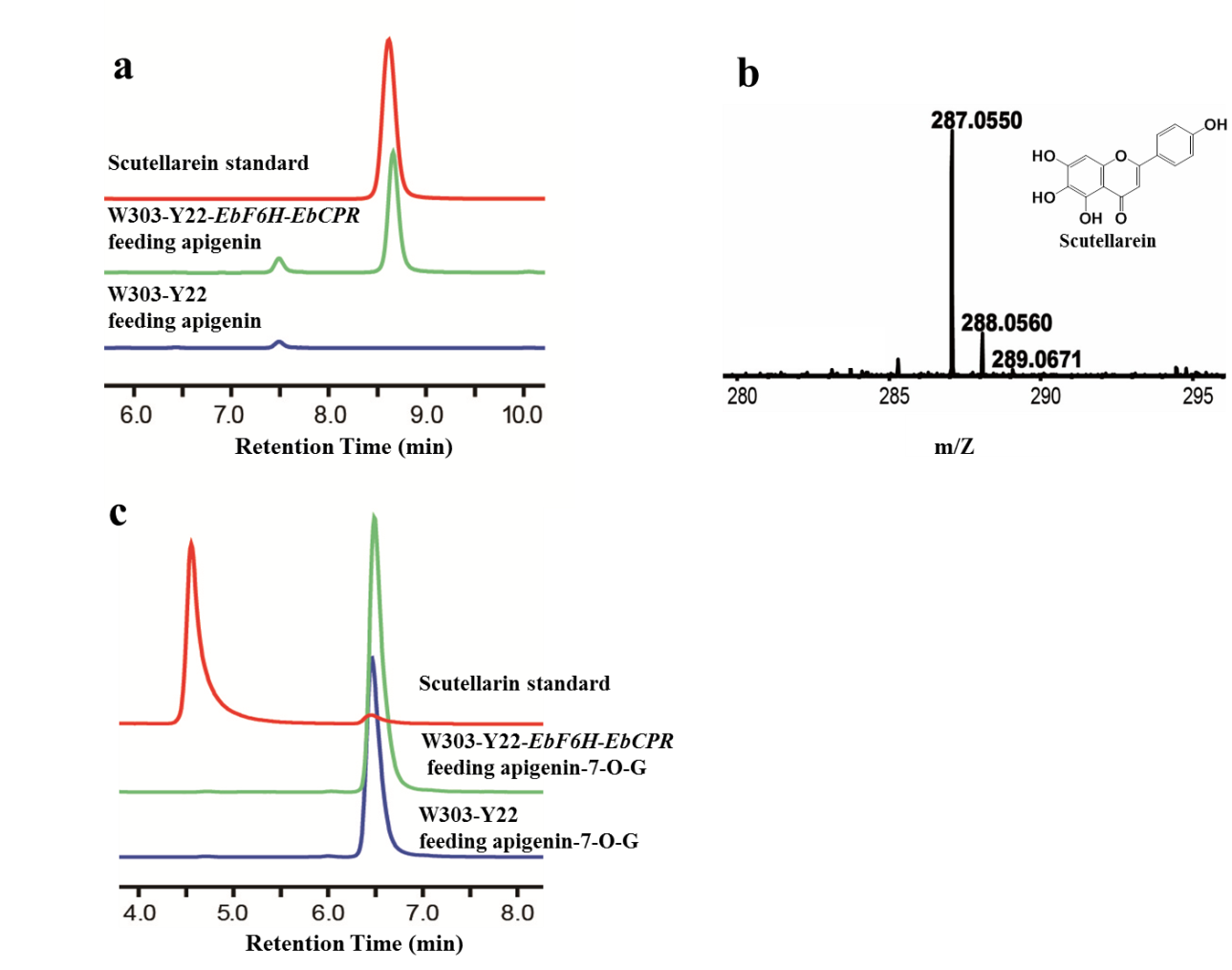


**Supplementary Figure 6 | Determining the priority of glycosylation and hydroxylation.** (**a**) HPLC analysis of the fermentative products of W303-Y22-*EbF6H*-*EbCPR* feeding apigenin as substrate. W303 harboring empty vector was used as the negative control. (**b**) MS analysis of scutellarein in the fermented products of W303-Y22-*EbF6H*-*EbCPR* by feeding apigenin as substrate. (**c**) HPLC analysis of the fermented products of W303-Y22-*EbF6H*-*EbCPR* and W303-Y22 by feeding apigenin-7-O-glucuronide as substrate.


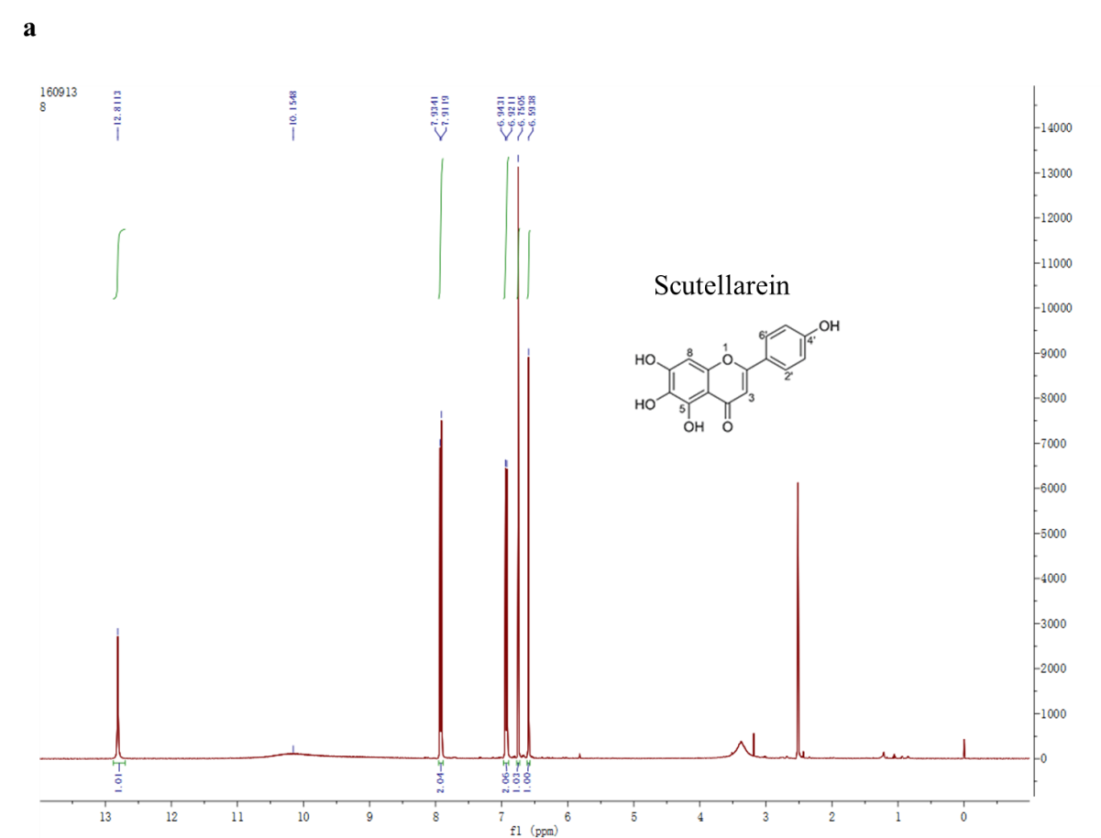


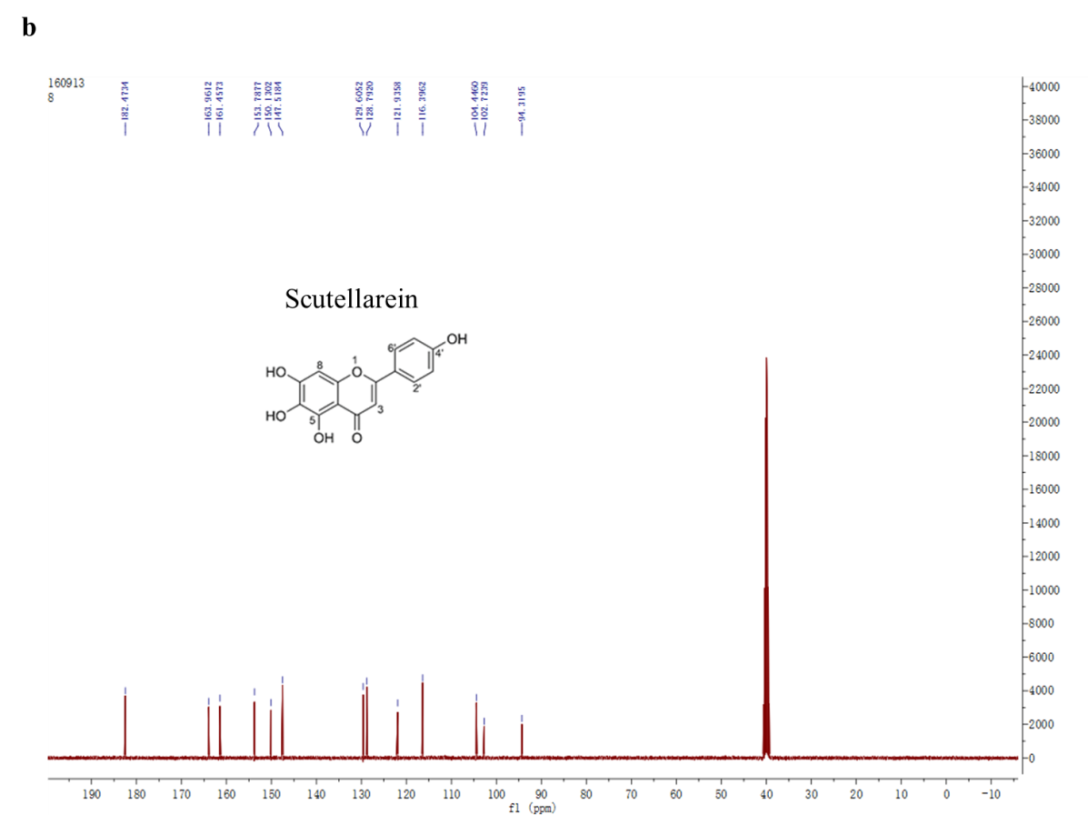


**Supplementary Figure 7 | NMR (nuclear magnetic resonance) identification of scutellarein structure.** (**a**) ^1^H NMR spectrum of scutellarein. (**b**) ^13^C NMR spectrum of scutellarein.


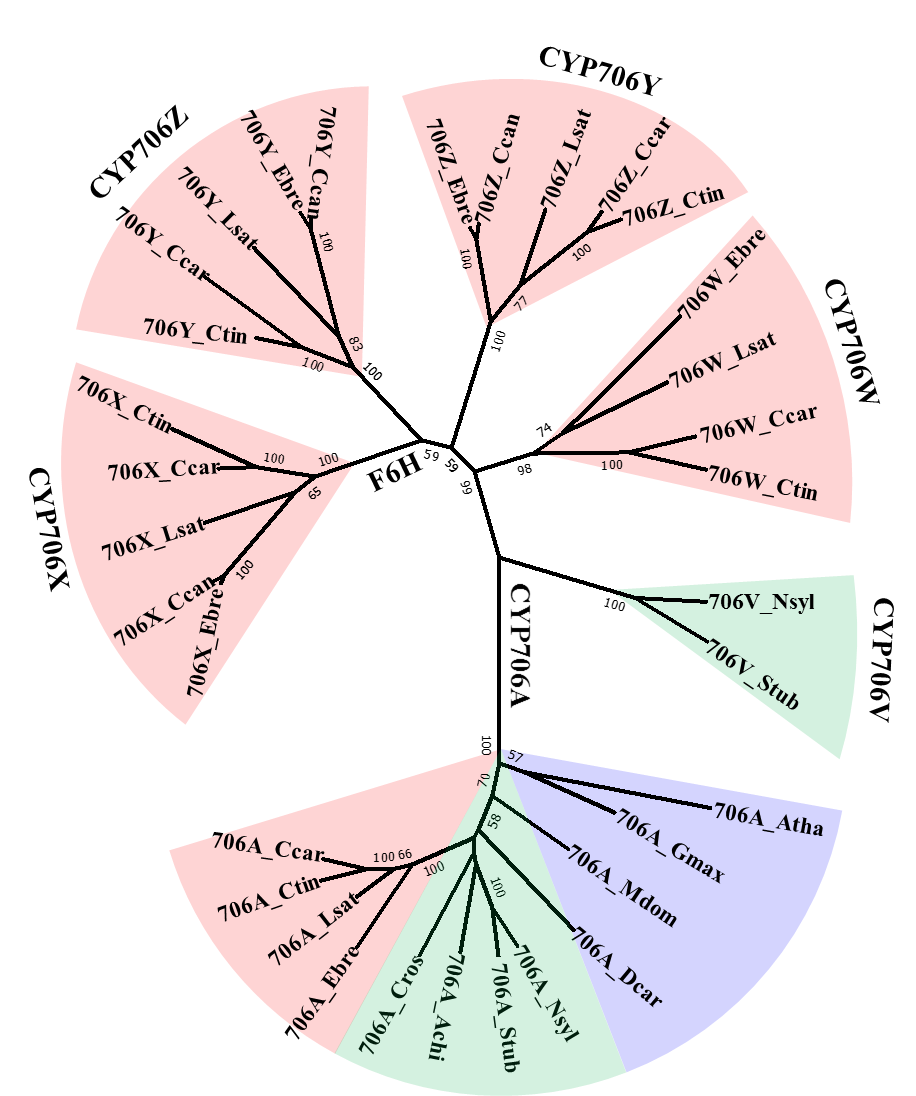


**Supplementary Figure 8 |** **The phylogenetic relationships of P450 CYP706 family.** The maximum-likelihood tree was constructed by 33 CYP706 family genes distributed in six subfamilies, *CYP706A*, *CYP706V*, *CYP706W, CYP706Z, CYP706Y*, and *CYP706X* (Supplementary Data 4). The *CYP706X* has potential function for flavone-6-hydroxylase.


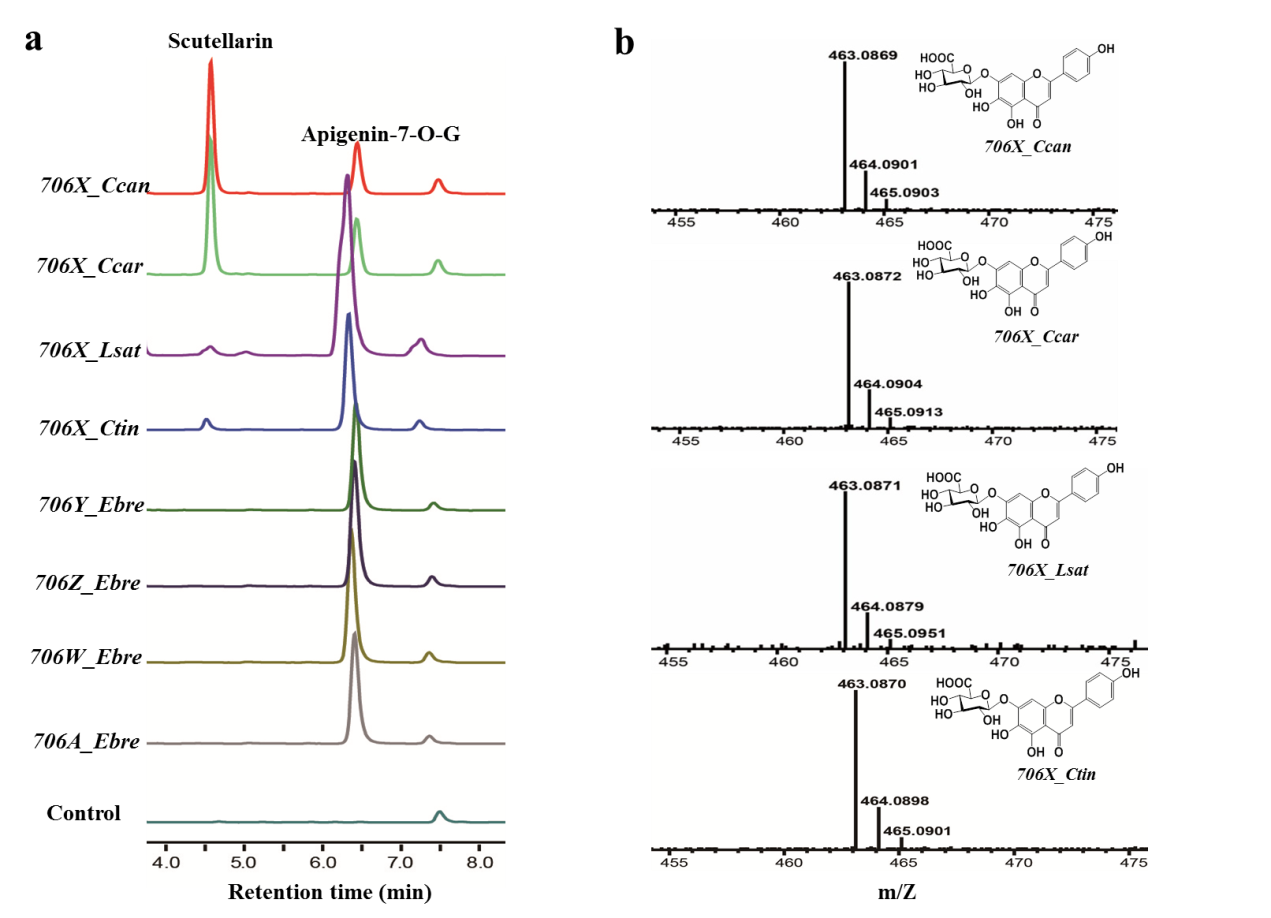


**Supplementary Figure 9 | Fermentation products of different P450 candidate genes from CYP706 family.** (**a**) HPLC analysis of fermented products of strains with different P450 candidate genes from CYP706 family. The analysis included four 706X genes in other Asteraceae genomes (*Conyza Canadensis*, *Cynara cardunculus,* *Lactuca sativa* and *Carthamus tinctorius*), and four genes (i.e., 706A, 706W, 706Z, 706Y) from other subfamilies in *E. breviscapus.* (**b**) MS analysis of scutellarin in the fermented products of CcF6H (*706X_Ccan*), CcarF6H (*706X_Ccar*), LsF6H (*706X_Lsat*) and CtF6H (*706X_Ctin*).

**Supplementary Table 1 | The copy numbers of the candidate genes for apigenin biosynthesis in thirteen plants**

| **Species** | **PAL** | **C4H** | **4CL** | **CHS** | **CHI** | **FSII** | **FSI** |
| --- | --- | --- | --- | --- | --- | --- | --- |
| *Erigeron breviscapus* | 5 | 2 | 2 | 4 | 1 | 1 | 0 |
| *Conyza canadensis* | 5 | 4 | 2 | 5 | 1 | 1 | 0 |
| *Lactuca sativa* | 3 | 3 | 2 | 7 | 1 | 1 | 0 |
| *Cynara cardunculus* | 5 | 2 | 2 | 3 | 2 | 1 | 0 |
| *Carthamus tinctorius* | 3 | 1 | 2 | 1 | 1 | 1 | 0 |
| *Daucus carota* | 4 | 2 | 3 | 11 | 1 | 0 | 1 |
| *Actinidia chinensis* | 8 | 3 | 3 | 6 | 3 | 0 | 0 |
| *Catharanthus roseus* | 3 | 1 | 2 | 4 | 1 | 1 | 0 |
| *Nicotiana sylvestris* | 4 | 2 | 4 | 4 | 1 | 0 | 0 |
| *Solanum tuberosum* | 8 | 2 | 4 | 8 | 2 | 0 | 0 |
| *Malus domestica* | 4 | 1 | 2 | 10 | 8 | 0 | 0 |
| *Glycine max* | 9 | 2 | 9 | 15 | 3 | 2 | 0 |
| *Arabidopsis thaliana* | 4 | 1 | 4 | 1 | 1 | 0 | 0 |

**PAL:** phenylalanine ammonia lyase; **C4H:** cinnamate 4-hydroxylase; **4CL:** 4-coumaroyl-CoA ligase; **CHS:** chalcone synthase; **CHI:** chalcone isomerase; **FSII:** flavone synthase II; **FSI:** flavone synthase I

**Supplementary Table 2 | The candidate genes for biosynthesis apigenin in *E. breviscapus***

| **Pathway_Genes** | **Gene_ID** | **Wild (RPKM)** | **Cultivation(RPKM)** |
| --- | --- | --- | --- |
| EbrePAL1* | Ebre_g007673 | 706.9 | 136.5 |
| EbrePAL2 | Ebre_g070226 | 100.1 | 21.5 |
| EbrePAL3 | Ebre_g030573 | 169.3 | 24.6 |
| EbrePAL4 | Ebre_g003237 | 0.0 | 0.0 |
| EbrePAL5 | Ebre_g027826 | 0.0 | 0.0 |
| EbreC4H1* | Ebre_g015385 | 187.0 | 97.0 |
| EbreC4H2 | Ebre_g001341 | 25.5 | 17.4 |
| Ebre4CL1* | Ebre_g019519 | 185.1 | 80.7 |
| Ebre4CL2 | Ebre_g033426 | 15.1 | 5.5 |
| EbreCHS1* | Ebre_g037634 | 787.7 | 989.6 |
| EbreCHS2 | Ebre_g037444 | 0.0 | 0.0 |
| EbreCHS3 | Ebre_g038690 | 1.2 | 0.4 |
| EbreCHS4 | Ebre_g036606 | 0.0 | 0.0 |
| EbreCHI* | Ebre_g036111 | 51.1 | 43.5 |
| EbreFSII* | Ebre_g059123 | 151.3 | 156.6 |

* : indicates the highly expressed copies which were used to construct the biosynthetic pathway of apigenin in yeast.

RPKM: Reads Per Kilobase per Million mapped reads.

**Supplementary Table 3 | Plasmids used in this study**

| **Plasmids** | **Relevant characteristics** | **Source** |
| --- | --- | --- |
| T1-(*tTPI1-tPGI*) | pMD-19T, *AmpR*, *tTPI1*, *tPGI*, Homologous arm L1and L2 | Li *et al.^5^* |
| T2-(*tADH1-tCYC1*) | pMD-19T, *AmpR*, *tADH1*, *tCYC1*, Homologous arm L2and L3 | Li *et al.^5^* |
| T3-(*tFBA1-tPDC1*) | pUC57, *KanR*, *tFBA1*, *tPDC1*, Homologous arm L3and L4 | Li *et al.^5^* |
| T4-(*tRPS2-tTDH1*) | pUC57, *KanR*, *tRPS2*, *tTDH1*, Homologous arm L4 and L5 | Li *et al.^5^* |
| T5-(*tCCW12-tRPL9A*) | pUC57, *KanR*, *tCCW12*, *tRPL9A*, Homologous arm L5 and L6 | Li *et al.^5^* |
| YCplac22 | Centromeric vector, *TRP1*, *AmpR* | Gietz *et al.* ^6^ |
| YCplac33 | Centromeric vector, *URA3*, *AmpR* | Gietz *et al.*^6^ |
| YCplac22-PE | Centromeric vector, *TRP1*, *AmpR*, *pPGK1* and *tCYC1* | GENEWIZ |
| YCplac33-PE | Centromeric vector, *URA3*, *AmpR*, *pPGK1* and *tCYC1* | GENEWIZ |
| YCplac22-TC | Centromeric vector, *TRP1*, *AmpR*, *tGPD1* and *tPFK1* | GENEWIZ |
| YCplac33-GP | Centromeric vector, *URA3*, *AmpR*, *tTDH1* and *tCYC1* | GENEWIZ |
| T1-*Eb4CL*-*EbCHS* | T1 vector, *pADH1*-*Eb4CL*-*tTPI1*/*pHXT7*-*EbCHS*-*tPGI* | This study |
| T2-*EbCHI*-*EbFSII* | T2 vector, *pPGI*-*EbCHI*-*tADH1*/*pRPL8A*-*EbFSII*-*tCYC1* | This study |
| T3-*EbPAL*-*EbC4H* | T3 vector, *pADH1*-*EbPAL*-*tFBA1*/*pTDH3*-*EbC4H*-*tPDC1* | This study |
| T4-*ACS_Se_^P641L^*-*ALD6* | T4 vector, *pTEF2*-*ACS_Se_^L641P^*-*tRPS2*/*pPGK1-ALD6-tTDH1* | This study |
| T5-*ADH2* | T5 vector, *pHXT7*-*ADH2*-*tRPL19A* | This study |
| Y22-*ObF6H*-*EbCPR* | YCplac22 vector, *pPGK1*-*ObF6H*-*tCYC1*/*pTDH3*-*EbCPR*-*tTDH1* | This study |
| Y22-*EbF6H*-*EbCPR* | YCplac22 vector, *pPGK1*-*EbF6H*-*tCYC1*/*pTDH3*-*EbCPR*-*tTDH1* | This study |
| Y33-*EbF7GAT*-*EbUDPGDH* | YCplac33 vector, *pADH1*-*EbF7GAT*-*tGPD1*/*pTDH3*-*EbUDPGDH*-*tPFK1* | This study |
| pET28a | pBR322 ori with *pT7*; *KanR* | Novagen |
| pET28a-*EbF7GAT* | pET28a vector, *Nde*І*-EbF7GAT-Xho*I | This study |
| Y33-*EbF7GAT* | YCplac33 vector, *pPGK1*-*EbF7GAT*-*tCYC1* | This study |
| Y22-*EbCPR* | YCplac22 vector, *pPGK1*-*EbCPR*-*tCYC1* | This study |
| Y22-*CcF6H*-*EbCPR* | YCplac22 vector, *pPGK1*-*CcF6H*-*tCYC1*/*pTDH3*-*EbCPR*-*tTDH1* | This study |
| Y22-*CcarF6H*-*EbCPR* | YCplac22 vector, *pPGK1*-*CcarF6H*-*tCYC1*/*pTDH3*-*EbCPR*-*tTDH1* | This study |
| Y22-*CtF6H*-*EbCPR* | YCplac22 vector, *pPGK1*-*CtF6H*-*tCYC1*/*pTDH3*-*EbCPR*-*tTDH1* | This study |
| Y22-*LsF6H*-*EbCPR* | YCplac22 vector, *pPGK1*-*LsF6H*-*tCYC1*/*pTDH3*-*EbCPR*-*tTDH1* | This study |
| Y22-*067055*-*EbCPR* | YCplac22 vector, *pPGK1*-Ebre_g067055-*tCYC1*/*pTDH3*-*EbCPR*-*tTDH1* | This study |
| Y22-*037901*-*EbCPR* | YCplac22 vector, *pPGK1*-Ebre_g037901-*tCYC1*/*pTDH3*-*EbCPR*-*tTDH1* | This study |
| Y22-*048520*-*EbCPR* | YCplac22 vector, *pPGK1*-Ebre_g048520-*tCYC1*/*pTDH3*-*EbCPR*-*tTDH1* | This study |
| Y22-*007711*-*EbCPR* | YCplac22 vector, *pPGK1*-Ebre_g007711-*tCYC1*/*pTDH3*-*EbCPR*-*tTDH1* | This study |
| Y22-*030906*-*EbCPR* | YCplac22 vector, *pPGK1*-Ebre_g030906-*tCYC1*/*pTDH3*-*EbCPR*-*tTDH1* | This study |
| Y22-*056920*-*EbCPR* | YCplac22 vector, *pPGK1*-Ebre_g056920-*tCYC1*/*pTDH3*-*EbCPR*-*tTDH1* | This study |
| Y22-*029091*-*EbCPR* | YCplac22 vector, *pPGK1*-Ebre_g029091-*tCYC1*/*pTDH3*-*EbCPR*-*tTDH1* | This study |
| Y22-*043487*-*EbCPR* | YCplac22 vector, *pPGK1*-Ebre_g043487-*tCYC1*/*pTDH3*-*EbCPR*-*tTDH1* | This study |
| Y22-*004063*-*EbCPR* | YCplac22 vector, *pPGK1*-Ebre_g004063-*tCYC1*/*pTDH3*-*EbCPR*-*tTDH1* | This study |
| Y22-*032678*-*EbCPR* | YCplac22 vector, *pPGK1*-Ebre_g032678-*tCYC1*/*pTDH3*-*EbCPR*-*tTDH1* | This study |
| Y22-*052890*-*EbCPR* | YCplac22 vector, *pPGK1*-Ebre_g052890-*tCYC1*/*pTDH3*-*EbCPR*-*tTDH1* | This study |
| Y22-*024112*-*EbCPR* | YCplac22 vector, *pPGK1*-Ebre_g024112-*tCYC1*/*pTDH3*-*EbCPR*-*tTDH1* | This study |
| Y22-*065571*-*EbCPR* | YCplac22 vector, *pPGK1*-Ebre_g065571-*tCYC1*/*pTDH3*-*EbCPR*-*tTDH1* | This study |
| Y22-*033640*-*EbCPR* | YCplac22 vector, *pPGK1*-Ebre_g033640-*tCYC1*/*pTDH3*-*EbCPR*-*tTDH1* | This study |
| Y22-*055990*-*EbCPR* | YCplac22 vector, *pPGK1*-Ebre_g055990-*tCYC1*/*pTDH3*-*EbCPR*-*tTDH1* | This study |
| Y22-*011872*-*EbCPR* | YCplac22 vector, *pPGK1*-Ebre_g011872-*tCYC1*/*pTDH3*-*EbCPR*-*tTDH1* | This study |
| Y22-011873-*EbCPR* | YCplac22 vector, *pPGK1*-Ebre_g011873-tCYC1/*pTDH3*-*EbCPR*-*tTDH1* | This study |
| Y22-*017226*-*EbCPR** | YCplac22 vector, *pPGK1*-Ebre_g017226-*tCYC1*/*pTDH3*-*EbCPR*-*tTDH1* | This study |
| Y22-*028901*-*EbCPR* | YCplac22 vector, *pPGK1*-Ebre_g028901-*tCYC1*/*pTDH3*-*EbCPR*-*tTDH1* | This study |
| Y22-*016500*-*EbCPR* | YCplac22 vector, *pPGK1*-Ebre_g016500-*tCYC1*/*pTDH3*-*EbCPR*-*tTDH1* | This study |
| Y22-*048172*-*EbCPR* | YCplac22 vector, *pPGK1*-Ebre_g048172-*tCYC1*/*pTDH3*-*EbCPR*-*tTDH1* | This study |
| Y22-*051967*-*EbCPR* | YCplac22 vector, *pPGK1*-Ebre_g051967-*tCYC1*/*pTDH3*-*EbCPR*-*tTDH1* | This study |
| Y22-*061651*-*EbCPR* | YCplac22 vector, *pPGK1*-Ebre_g061651-*tCYC1*/*pTDH3*-*EbCPR*-*tTDH1* | This study |
| Y22-*061652*-*EbCPR* | YCplac22 vector, *pPGK1*-Ebre_g061652-*tCYC1*/*pTDH3*-*EbCPR*-*tTDH1* | This study |
| Y22-*002073*-*EbCPR* | YCplac22 vector, *pPGK1*-Ebre_g002073-*tCYC1*/*pTDH3*-*EbCPR*-*tTDH1* | This study |
| Y22-*038429*-*EbCPR* | YCplac22 vector, *pPGK1*-Ebre_g038429-*tCYC1*/*pTDH3*-*EbCPR*-*tTDH1* | This study |
| Y22-*026712*-*EbCPR* | YCplac22 vector, *pPGK1*-Ebre_g026712-*tCYC1*/*pTDH3*-*EbCPR*-*tTDH1* | This study |
| Y22-*043846*-*EbCPR* | YCplac22 vector, *pPGK1*-Ebre_g043846-*tCYC1*/*pTDH3*-*EbCPR*-*tTDH1* | This study |
| Y22-*005573*-*EbCPR* | YCplac22 vector, *pPGK1*-Ebre_g005573-*tCYC1*/*pTDH3*-*EbCPR*-*tTDH1* | This study |
| Y22-*028301*-*EbCPR* | YCplac22 vector, *pPGK1*-Ebre_g028301-*tCYC1*/*pTDH3*-*EbCPR*-*tTDH1* | This study |
| Y22-*047827*-*EbCPR* | YCplac22 vector, *pPGK1*-Ebre_g047827-*tCYC1*/*pTDH3*-*EbCPR*-*tTDH1* | This study |
| Y22-*050362*-*EbCPR* | YCplac22 vector, *pPGK1*-Ebre_g050362-*tCYC1*/*pTDH3*-*EbCPR*-*tTDH1* | This study |
| Y22-*028780*-*EbCPR* | YCplac22 vector, *pPGK1*-Ebre_g028780-*tCYC1*/*pTDH3*-*EbCPR*-*tTDH1* | This study |
| Y22-*041463*-*EbCPR* | YCplac22 vector, *pPGK1*-Ebre_g041463-*tCYC1*/*pTDH3*-*EbCPR*-*tTDH1* | This study |
| Y22-*049752*-*EbCPR* | YCplac22 vector, *pPGK1*-Ebre_g049752-*tCYC1*/*pTDH3*-*EbCPR*-*tTDH1* | This study |
| Y22-*035430*-*EbCPR* | YCplac22 vector, *pPGK1*-Ebre_g035430-*tCYC1*/*pTDH3*-*EbCPR*-*tTDH1* | This study |
|  |  |  |

* : indicates *EbF6H*.

S**upplementary Table 4 | Strains used in this study**

| **Strains** | **Relevant characteristics** | **Source** |
| --- | --- | --- |
| W303-1B | *W303* *(MATα, ade2–1; ura3–1; his3–11,15; trp1–1; leu2–3,112; can 1–100)* | Thomas (1989)^7^ |
| BL21 (DE3) | *F-; ompT; hsdS（rBB-mB－);gal;dcm（DE3）* | TransGen |
| FE189 | BL21(DE3) carrying pET28a-*EbF7GAT* | This study |
| SC1 | W303 *YORW△17*::*his3*/*pADH1*-*Eb4CL*-TPI1t/*pHXT7*-*EbCHS*- *tTPG1*/*pPGI1* -*EbCHI*-*tADH1*/*pRPL8A*-*EbFSII*-*tCYC1*/*pADH1*-*EbPAL*-*tFBA1*/*pTDH3*-*EbC4H*-*tPDC1* | This study |
| SC1-Y22 | SC1 carrying YCplac22 | This study |
| SC1-Y22-Y33 | SC1 carrying YCplac22and YCplac33 | This study |
| SC1-FU | SC1 carrying Y33-*EbF7GAT*-*EbUDPGDH* | This study |
| W303-F | W303 carrying Y33- *EbF7GAT* | This study |
| SC1-FC | SC1 carrying Y22-*EbF6H*-*EbCPR* | This study |
| SC1-FU-FC | SC1 carrying Y22-*EbF6H*-*EbCPR* and Y33-*EbF7GAT*-*EbUDPGDH* | This study |
| W303-Y22 | W303 carrying YCplac22 | This study |
| W303-C | W303 carrying Y22-*EbCPR* | This study |
| W303-FC | W303 carrying Y22-*EbF6H*-*EbCPR* | This study |
| SC1-FU-CcarC | SC1 carrying Y22-*CcarF6H*-*EbCPR* and Y33-*EbF7GAT*-*EbUDPGDH* | This study |
| SC1-FU-CcC | SC1 carrying Y22-*CcF6H*-*EbCPR* and Y33-*EbF7GAT*-*EbUDPGDH* | This study |
| SC1-FU-CtC | SC1 carrying Y22-*CtF6H*-*EbCPR* and Y33-*EbF7GAT*-*EbUDPGDH* | This study |
| SC1-FU-LsC | SC1 carrying Y22-*LsF6H*-*EbCPR* and Y33-*EbF7GAT*-*EbUDPGDH* | This study |
| SC1-F | SC1 carrying Y33-*EbF7GAT* | This study |
| △M-FU-FC | SC1-FU-FC△*mls1* | This study |
| △MC-FU-FC | SC1-FU-FC△*mls1*△*cit2* | This study |
| △MC-FU-FC-AAA | △MC-FU-FC *YORW△15::leu2/pTEF2-ACS_Se_^L641P^-tRPS2/pPGK1-ALD6-tTDH1/pHXT7*-*ADH2*-*tRPL19A* | This study |
| S1 | SC1 carrying Y22-*067055*-*EbCPR* and Y33-*EbF7GAT*-*EbUDPGDH* | This study |
| S2 | SC1 carrying Y22-*037901*-*EbCPR* and Y33-*EbF7GAT*-*EbUDPGDH* | This study |
| S3 | SC1 carrying Y22-*048520*-*EbCPR* and Y33-*EbF7GAT*-*EbUDPGDH* | This study |
| S4 | SC1 carrying Y22-*007711*-*EbCPR* and Y33-*EbF7GAT*-*EbUDPGDH* | This study |
| S5 | SC1 carrying Y22-*030906*-*EbCPR* and Y33-*EbF7GAT*-*EbUDPGDH* | This study |
| S6 | SC1 carrying Y22-*056920*-*EbCPR* and Y33-*EbF7GAT*-*EbUDPGDH* | This study |
| S7 | SC1 carrying Y22-*029091*-*EbCPR* and Y33-*EbF7GAT*-*EbUDPGDH* | This study |
| S8 | SC1 carrying Y22-*043487*-*EbCPR* and Y33-*EbF7GAT*-*EbUDPGDH* | This study |
| S9 | SC1 carrying Y22-*004063*-*EbCPR* and Y33-*EbF7GAT*-*EbUDPGDH* | This study |
| S10 | SC1 carrying Y22-*032678*-*EbCPR* and Y33-*EbF7GAT*-*EbUDPGDH* | This study |
| S11 | SC1 carrying Y22-*052890*-*EbCPR* and Y33-*EbF7GAT*-*EbUDPGDH* | This study |
| S12 | SC1 carrying Y22-*024112*-*EbCPR* and Y33-*EbF7GAT*-*EbUDPGDH* | This study |
| S13 | SC1 carrying Y22-*065571*-*EbCPR* and Y33-*EbF7GAT*-*EbUDPGDH* | This study |
| S14 | SC1 carrying Y22-*033640*-*EbCPR* and Y33-*EbF7GAT*-*EbUDPGDH* | This study |
| S15 | SC1 carrying Y22-*055990*-*EbCPR* and Y33-*EbF7GAT*-*EbUDPGDH* | This study |
| S16 | SC1 carrying Y22-*011872*-*EbCPR* and Y33-*EbF7GAT*-*EbUDPGDH* | This study |
| S17 | SC1 carrying Y22-011873-*EbCPR* and Y33-*EbF7GAT*-*EbUDPGDH* | This study |
| S18 | SC1 carrying Y22-*017226*-*EbCPR* and Y33-*EbF7GAT*-*EbUDPGDH* | This study |
| S19 | SC1 carrying Y22-*028901*-*EbCPR* and Y33-*EbF7GAT*-*EbUDPGDH* | This study |
| S20 | SC1 carrying Y22-*016500*-*EbCPR* and Y33-*EbF7GAT*-*EbUDPGDH* | This study |
| S21 | SC1 carrying Y22-*048172*-*EbCPR* and Y33-*EbF7GAT*-*EbUDPGDH* | This study |
| S22 | SC1 carrying Y22-*051967*-*EbCPR* and Y33-*EbF7GAT*-*EbUDPGDH* | This study |
| S23 | SC1 carrying Y22-*061651*-*EbCPR* and Y33-*EbF7GAT*-*EbUDPGDH* | This study |
| S24 | SC1 carrying Y22-*061652*-*EbCPR* and Y33-*EbF7GAT*-*EbUDPGDH* | This study |
| S25 | SC1 carrying Y22-*002073*-*EbCPR* and Y33-*EbF7GAT*-*EbUDPGDH* | This study |
| S26 | SC1 carrying Y22-*038429*-*EbCPR* and Y33-*EbF7GAT*-*EbUDPGDH* | This study |
| S27 | SC1 carrying Y22-*026712*-*EbCPR* and Y33-*EbF7GAT*-*EbUDPGDH* | This study |
| S28 | SC1 carrying Y22-*043846*-*EbCPR* and Y33-*EbF7GAT*-*EbUDPGDH* | This study |
| S29 | SC1 carrying Y22-*005573*-*EbCPR* and Y33-*EbF7GAT*-*EbUDPGDH* | This study |
| S30 | SC1 carrying Y22-*028301*-*EbCPR* and Y33-*EbF7GAT*-*EbUDPGDH* | This study |
| S31 | SC1 carrying Y22-*047827*-*EbCPR* and Y33-*EbF7GAT*-*EbUDPGDH* | This study |
| S32 | SC1 carrying Y22-*050362*-*EbCPR* and Y33-*EbF7GAT*-*EbUDPGDH* | This study |
| S33 | SC1 carrying Y22-*028780*-*EbCPR* and Y33-*EbF7GAT*-*EbUDPGDH* | This study |
| S34 | SC1 carrying Y22-*041463*-*EbCPR* and Y33-*EbF7GAT*-*EbUDPGDH* | This study |
| S35 | SC1 carrying Y22-*049752*-*EbCPR* and Y33-*EbF7GAT*-*EbUDPGDH* | This study |
| S36 | SC1 carrying Y22-*035430*-*EbCPR* and Y33-*EbF7GAT*-*EbUDPGDH* | This study |

**Supplementary Table 5 | Kinetic parameters of EbF7GAT towards apigenin and scutellarein**

| **Substrates** | **Km(uM)** | **Kcat(S^-1^)** | **kcat/Km(S^-1^M^-1^)** |
| --- | --- | --- | --- |
| Apigenin | 9.24±3.015 | 0.57±0.083 | 6.17×10^4^ |
| Scutellarein | 70.15±25.97 | 0.24±0.027 | 3.42×10^3^ |

These data represent the average of three independent measurements ±Standard Deviation (SD).

**Supplementary Table 6 | Conversion rates of different substrates catalyzed by EbF7GAT**

| **Substrates** | **Conversion rate** |
| --- | --- |
| Narigenin | 95.05% |
| Kaempferol | 99.29% |
| Quercetin | 100.00% |
| Chrysin | 67.35% |
| Baicalein | 88.26% |
| Luteolin | 98.47% |
| Diosmetin | 60.71% |
| Chrysoeriol | 96.00% |
| Apigenin | 89.50% |
| Scutellarein | 99.74% |

**Supplementary Table 7 | The distribution proportion of different flavonoid products**

| **Compound** | **Intracellular** | **Fermented supernatant** |
| --- | --- | --- |
| Apigenin | 90.9% | 9.1% |
| Scutellarein | 98.7% | 1.3% |
| Scutellarin | 77.9% | 22.1% |
| Apigenin-7-O-glucuronide | 26.6% | 73.4% |

**Supplementary Table 8 | The function identification of other species orthologous genes in *E. breviscapus***

| **Other species F6H** | **Orthologous genes (Best hit)** | **Scutellarin production** |
| --- | --- | --- |
| ObF6H | Ebre_g050676 | N.D. |
| MpF6H | Ebre_g050676 | N.D. |
| SmF6H | Ebre_g050676 | N.D. |
| SbF6H | Ebre_g050676 | N.D. |
| GmF6H | Ebre_g047942 | N.D. |

**Ob:** *Ocimum basilicum*; **Mp:** *Mentha piperita*; **Sm:** *Salvia miltiorrhiza*; **Gm:** *Glycine max*; **Sb:** *Scutellaria baicalensis*. The accession numbers are AGF30364 (ObF6H), AGF30366 (MpF6H), AJD25201 (SmF6H), CAA71514 (GmF6H), MF363006 (SbF6H). '**N.D.**' represented that scutellarin was not detected.

**Supplementary Table 9 | The number of P450 genes in thirteen plant genomes**

| **Species** | **Numb. of P450 Genes** |
| --- | --- |
| *Actinidia chinensis* | 231 |
| *Arabidopsis thaliana* | 274 |
| *Carthamus tinctorius* | 190 |
| *Catharanthus roseus* | 180 |
| *Conyza canadensis* | 249 |
| *Daucus carota* | 387 |
| *Erigeron breviscapus* | 312 |
| *Cynara cardunculus* | 263 |
| *Glycine max* | 342 |
| *Lactuca sativa* | 253 |
| *Malus domestica* | 347 |
| *Nicotiana sylvestris* | 251 |
| *Solanum tuberosum* | 384 |

**Supplementary Table 10 | Primers used in vectors construction**

| **Primer Name** | **Primer Sequence (5’→3’)** |
| --- | --- |
| Eb4CL-F | 5’-ACCAGGTCTCAGATGGACTCTCAAAAGGAAATCATCTTCAGATCT-3’ |
| Eb4CL-R | 5’-ACCAGGTCTCAAGGTTTAAGATGGAACACCAGCAGCCA-3’ |
| EbCHS-F | 5’-ACCAGGTCTCAGAATGGCTTCTTCTATCGACATCGCTGC-3’ |
| EbCHS-R | 5’-ACCAGGTCTCACGATTTAAGTAGCGATAGCAGTAGTAGTTGGCAAAGAGT-3’ |
| EbCHI-F | 5’-ACCAGGTCTCAAGGTTTACAAACCGTACTTAGAAACGTCAGC-3’ |
| EbCHI-R | 5’-ACCAGGTCTCAGATGGCTGCTACTACTACTCCATTGAC-3’ |
| EbFSII-F | 5’-ACCAGGTCTCAGAATGAACATGTTGCAAGTTTTCCAATC-3’ |
| EbFSII-R | 5’-ACCAGGTCTCACGATTTAAGTAGAACCCAAGATTTGAGAACAG-3’ |
| EbPAL-F | 5’-CCGGTCTCAGAATATGGAAAACGGTCACGCTAACGGTG-3’ |
| EbPAL-R | 5’-CCGGTCTCAAGGTTTAACAGATTGGCAATGGAGCACCGTTCCAAC-3’ |
| EbC4H-F | 5’-CCGGTCTCAGATGATGGACTTGTTGTTGTTGGAAAAGA-3’ |
| EbC4H-R | 5’-CCGGTCTCACGATTTAGAAAGATCTTGGCTTAGCAACGATA-3’ |
| ACS_Se_^L641P^-F | 5’-CCGGTCTCAGAATATGTCTCAAACTCACAAGCACGC-3’ |
| ACS_Se_^L641P^-R | 5’-CCGGTCTCAAGGTTTAAGATGGCATAGCGATAGCTTGCTT-3’ |
| ALD6-F | 5’-CCGGTCTCAGATGATGACTAAGCTACACTTTGAC-3’ |
| ALD6-R | 5’-CCGGTCTCACGATTTACAACTTAATTCTGACAGCT-3’ |
| ADH2-F | 5’-CCCGGTCTCAGATGTCTATTCCAGAAACTCAA-3’ |
| ADH2-R | 5’-CCCGGTCTCACGATTTATTTAGAAGTGTCAACAACG-3’ |
| T1-F | 5’-CCCGGTCTCAATCGAACAAATCGCTCTTAA-3’ |
| T1-R | 5’-CCCGGTCTCACATCTGTATATGAGATAGTTGATTGTATGCTTGG-3’ |
| T2-F | 5’-GCTTGGTCTCAATCGTCATGTA-3’ |
| T2-R | 5’-CGAATTCGGTCTCAACCTAGTTATA-3’ |
| T3-F | 5’-GGGTCTCAATCGGAATTCGC-3’ |
| T3-R | 5’-GGGTCTCAACCTAAGCTTGTTAA-3’ |
| T4-F | 5’-GGGTCTCAATCGGAATTCATAAAGCAAT-3’ |
| T4-R | 5’-GGGGTCTCAACCTAAGCTTGCTTGTTGTC-3’ |
| T5-F | 5’-CCGGTCTCAACCTGCTTTTTATTTGATTTTGTG-3’ |
| T5-R | 5’-GGGGTCTCAATCGACTTAGTTTATTATTATTTA-3’ |
| pADH1+pHXT7-F | 5’-CCGGTCTCACATCTGTATATGAGATAGTTGATTG-3’ |
| pADH1+pHXT7-R | 5’-CCGGTCTCAATTCTTTTTGATTAAAATTAAAAAAACTTTT-3’ |
| pADH1+pHXT7-overlap-F | 5’-TCCGCCACCTACATGTTAGGAGTGGCAGCACGCTAATTCG-3’ |
| pADH1+pHXT7-overlap-R | 5’-CGAATTAGCGTGCTGCCACTCCTAACATGTAGGTGGCGGA-3’ |
| pPGI+pRPL8A-F | 5’-CCGGTCTCACATCAATGGGACGAAACAAATAGG-3’ |
| pPGI+pRPL8A-R | 5’-CCGGTCTCAATTCGGAGCTCGATGTGATAA-3’ |
| pPGI+pRPL8A-overlap-F | 5’-CGTTGATGTGTAGAAGTAGTGTGACCATGATTACGCCAAGCT-3’ |
| pPGI+pRPL8A-overlap-R | 5’-AGCTTGGCGTAATCATGGTCACACTACTTCTACACATCAACG-3’ |
| pADH1+pTDH3-F | 5’-CCGGTCTCAGAATTGTATATGAGATAGTTGATTGT-3’ |
| pADH1+pTDH3-R | 5’-CCGGTCTCACATCTTTGTTTGTTTATGTGTG-3’ |
| pADH1+pTDH3-overlap-F | 5’-CCCTCCGCCACCTACATGTATACTAGCGTTGAATGTTAGC-3’ |
| pADH1+pTDH3-overlap-R | 5’-GCTAACATTCAACGCTAGTATACATGTAGGTGGCGGAGGG-3’ |
| pTEF2+pPGK1-F | 5’-CCGGTCTCAATTCGGTACTAGTGTTTAGTTAAT-3’ |
| pTEF2+pPGK1-R | 5’-CCGGTCTCACATCTGTTTTATATTTGTTGTAAA-3’ |
| pTEF2+pPGK1-overlap-F | 5’-CGCTTGACATCTACTATATGTAAGTATAACGCACAGATATTATAACATCTG-3’ |
| pTEF2+pPGK1-overlap-R | 5’-CAGATGTTATAATATCTGTGCGTTATACTTACATATAGTAGATGTCAAGCG-3’ |
| pHXT7+pPGK1-F | 5’-CCCGGTCTCAATTCCGTTTTTTTGTTTTTATATTTGTTGT-3’ |
| pHXT7+pPGK1-R | 5’-CCCGGTCTCACATCTTTGTTTGTTTATGTGTGTTTATTC-3’ |
| pHXT7+pPGK1-overlap-F | 5’-GATGTTATAATATCTGTGCGTAGTGGCAGCACGCTAATTCGA-3’ |
| pHXT7+pPGK1-overlap-R | 5’-TCGAATTAGCGTGCTGCCACTACGCACAGATATTATAACATC-3’ |
| Y22-GG-5F | 5’-CCGGTCTCAATCGATAAAGCAATCTTGATGAGGA-3’ |
| Y22-GG-3R | 5’-CCGGTCTCAACCTCTAGAGGGCCGCATCATG-3’ |
| PGK+TDH3-GG-5F | 5’-CCGGTCTCACATCGTTTTTTTGTTTTTATATTTGTTGTAAAAAG-3’ |
| PGK+TDH3-GG-3R | 5’-CCGGTCTCAATTCTTTGTTTGTTTATGTGTGTTTATTCG-3’ |
| PGK1+TDH3-overlap-5F | 5’-TCTTGAGTTGAAGTCAGGAATCTAAAATAATACTAGCGTTGAATGTTAGCGTC-3’ |
| PGK+TDH3-overlap-3R | 5’-GACGCTAACATTCAACGCTAGTATTATTTTAGATTCCTGACTTCAACTCAAGA-3’ |
| EbCPR-GG-5F | 5’-CCGGTCTCAGAATGCAATCATCATCAAACTCAATAA-3’ |
| EbCPR-GG-3R | 5’-CCGGTCTCACGATTTACCAAACATCACGAAGGTATCT-3’ |
| CcF6H-GG-5F | 5’-CCGGTCTCAGATGATCATCAGCGACCTGGGC-3’ |
| CcF6H-GG-3R | 5’-CCGGTCTCAAGGTTTAGCTATACAGACTAACGTCTTTCAG-3’ |
| CcarF6H-GG-5F | 5’-CCGGTCTCAGATGGTGAGTCACGAACTGGCT-3’ |
| CcarF6H-GG-3R | 5’-CCGGTCTCAAGGTTTACATGTACAGACTCGGATCAG-3’ |
| LsF6H-GG-5F | 5’-CCGGTCTCAGATGGCAAGTAACTCTCACGG-3’ |
| LsF6H-GG-3R | 5’-CCGGTCTCAAGGTTTACATGTACAGACTCAGATCAATCAG-3’ |
| CtF6H-GG-5F | 5’-CCGGTCTCAGATGGAAGCACCGAATAGTCA-3’ |
| CtF6H-GG-3R | 5’-CCGGTCTCAAGGTTTACATATACAGACTTGCGTCATTCA-3’ |
| EbF7GAT-5F(NdeI） | 5’-GCACATCCCATATGGAAAACATTGTAGTGATGTTC-3’ |
| EbF7GAT-GG-3R(XhoI) | 5’-CCGCCTCGAGCTAGACTCGGGCCGATTTTAGTG-3’ |
| EbF7GAT-5F(SalI） | 5’-CCCGGGTCGACATGGAAAAC ATTGTAGTGATGTTC-3’ |
| EbF7GAT-GG-3R(XbaI) | 5’-CCCGGTCTAGACTAGACTCGGGCCGATTTTAGTG-3’ |
| Y33-GG-5F | 5’-GGCAGGTCTCAACCTATTTATTGGAGAAAGATAACATATCATACTTT-3’ |
| Y33-GG-3R | 5’-GGCAGGTCTCAATCGATGATTGCAATGAAAAGTTTAAGT-3’ |
| EbF7GAT-GG-5F | 5’-GGCGGTCTCAGAATGGAAAACATTGTAGTGATGTTC-3’ |
| EbF7GAT-GG-3R | 5’-GGTCTCAAGGTCTAGACTCGGGCCGATTTTAGTG-3’ |
| TDH3+ADH1-GG-5F | 5’-GGCGGTCTCACATCTTTGTTTGTTTATGTGTGTTTATTC-3’ |
| TDH3+ADH1-GG-3R | 5’-GGCGGTCTCATCTGTATATGAGATAGTTGATTGTATGCT-3’ |
| TDH3+ADH1-overlap-5F | 5’-GCTAACATTCAACGCTAGTATACATGTAGGTGGCGGAGGGGAG-3’ |
| TDH3+ADH1-overlap-3R | 5’-CTCCCCTCCGCCACCTACATGTATACTAGCGTTGAATGTTAGC-3’ |
| EbUDPGDH-GG-5F | 5’-GGGTCTCAGATGGTGAAGATCTGCTGCATTGGA-3’ |
| EbUDPGDH-GG-3R | 5’-GGGTCTCACGATTAGGCCACGGCAGGCATGTCC-3’ |

**Supplementary Table 11 | Primers used in P450 enzymes library construction**

| **Primer Name** | **Primer Sequence (5’→3’)** |
| --- | --- |
| CYC-F3 | 5’-TCATGTAATTAGTTATGTCA-3’ |
| MID-R3 | 5’-TACACTAGAAGAACAGTA-3’ |
| MID-F3 | 5’-TACTGTTCTTCTAGTGTA-3’ |
| PGK-R3 | 5’-TGTTTTTATATTTGTTGTAA-3’ |
| Ebre_g067055-5F | 5’-CCGGTCTCAGATGGATGTATCTATAGCTTTGCTGT-3’ |
| Ebre_g067055-3R | 5’-CCGGTCTCAAGGTCTAGTCGCCACCTTCTTTATTTCT-3’ |
| Ebre_g037901-5F | 5’-TTACAACAAATATAAAAACAATGATGGAGACATGGTTCATCATA-3’ |
| Ebre_g037901-3R | 5’-TGACATAACTAATTACATGATTAAGCCCTGGGTGAGATTTG-3’ |
| Ebre_g048520-5F | 5’-TTACAACAAATATAAAAACAATGGAAACATGGTTCATCAT-3’ |
| Ebre_g048520-3R | 5’-TGACATAACTAATTACATGATCAAATGGTTAACTTTTCGG-3’ |
| Ebre_g007711-5F | 5’-TTACAACAAATATAAAAACAATGGAAACATTGAGCAAA-3’ |
| Ebre_g007711-3R | 5’-TGACATAACTAATTACATGATCATAAGTTGCGTATAATCAA-3’ |
| Ebre_g030906-5F | 5’-TTACAACAAATATAAAAACAATGGACTTCATTTTCAACTCC-3’ |
| Ebre_g030906-3R | 5’-TGACATAACTAATTACATGATTAATTCTCTTTTTTGACCCAGA-3’ |
| Ebre_g056920-5F | 5’-TTACAACAAATATAAAAACAATGTTAGTTGAAGTTTCACT-3’ |
| Ebre_g056920-3R | 5’-TGACATAACTAATTACATGATTATTGCCTTTTCATGATTT-3’ |
| Ebre_g029091-5F | 5’-TTACAACAAATATAAAAACAATGATTTCAGAGCTTGGATATAAGAGTT-3’ |
| Ebre_g029091-3R | 5’-TGACATAACTAATTACATGACTATCCTATGTAGAGACTTTCATCAGACA-3’ |
| Ebre_g043487-5F | 5’-TTACAACAAATATAAAAACAATGGCACAAATAATTATTAATGATCATGG-3’ |
| Ebre_g043487-3R | 5’-TGACATAACTAATTACATGATCACATATAGAGGCTGATGTTGGG-3’ |
| Ebre_g004063-5F | 5’-TTACAACAAATATAAAAACAATGATAGGAACAGTGAGTACAATTG-3’ |
| Ebre_g004063-3R | 5’-TGACATAACTAATTACATGAGGCAAAGTTTTGCCCAATGC-3’ |
| Ebre_g032678-5F | 5’-TTACAACAAATATAAAAACAATGTTTGAAGTTATATCTATAATTCTAG-3’ |
| Ebre_g032678-3R | 5’-TGACATAACTAATTACATGATTATTGTTGCGATAAGCG-3’ |
| Ebre_g024112-5F | 5’-TTACAACAAATATAAAAACAATGACAACCCACATAGACACTC-3’ |
| Ebre_g024112-3R | 5’-TGACATAACTAATTACATGATCATGACAAACACGATGAAGTG-3’ |
| Ebre_g065571-5F | 5’-TTACAACAAATATAAAAACAATGGCTGCCAGTTTAACATCC-3’ |
| Ebre_g065571-3R | 5’-TGACATAACTAATTACATGATCAACTGACAACTGGATCAGCT-3’ |
| Ebre_g033640-5F | 5’-TTACAACAAATATAAAAACAATGGCTAACACCTATGGTCCC-3’ |
| Ebre_g033640-3R | 5’-TGACATAACTAATTACATGATCACATATAGAGGCTGATGTTGGG-3’ |
| Ebre_g055990-5F | 5’-TTACAACAAATATAAAAACAATGATATCAAGTAGTCAAGTTAGCCA-3’ |
| Ebre_g055990-3R | 5’-TGACATAACTAATTACATGAATTGGAACCCTTCAGGATTGTGTATC-3’ |
| Ebre_g011872-5F | 5’-TTACAACAAATATAAAAACAATGCTTCGAAACAAGCCCG-3’ |
| Ebre_g011872-3R | 5’-TGACATAACTAATTACATGATTAGCAAGTGGGATATAGATGGTGC-3’ |
| Ebre_g011873-5F | 5’-TTACAACAAATATAAAAACAATGTGTACTCTTTTAATTAGGTACAT-3’ |
| Ebre_g011873-3R | 5’-TGACATAACTAATTACATGATTAGCAAGTGGGATATAGATGG-3’ |
| Ebre_g017226-5F | 5’-TTACAACAAATATAAAAACAATGGCATCAAACGAGCTTGCTT-3’ |
| Ebre_g017226-3R | 5’-TGACATAACTAATTACATGATCACATGTAAAGGCTTAGGTCACTT-3’ |
| Ebre_g028901-5F | 5’-TTACAACAAATATAAAAACAATGGAAACATTGAGCAAAATTGT-3’ |
| Ebre_g028901-3R | 5’-TGACATAACTAATTACATGATCATAAGTTGCGTATAATCAAAGGA-3’ |
| Ebre_g016500-5F | 5’-TTACAACAAATATAAAAACAATGGAGAAGCGTCTAAGAGC-3’ |
| Ebre_g016500-3R | 5’-TGACATAACTAATTACATGATCAAACTCTACGTATATTTAAATGAG-3’ |
| Ebre_g048172-5F | 5’-TTACAACAAATATAAAAACAATGATCAAAGAAGTCTTGGCT-3’ |
| Ebre_g048172-3R | 5’-TGACATAACTAATTACATGACTAAAGTCCGTGTAGAATCAAATG-3’ |
| Ebre_g051967-5F | 5’-TTACAACAAATATAAAAACAATGGGAAGGATGCAAAATGAG-3’ |
| Ebre_g051967-3R | 5’-TGACATAACTAATTACATGATTAACCCTTAATTTTATGCAGGATC-3’ |
| Ebre_g052890-5F | 5’-TTACAACAAATATAAAAACAATGAGGGGAGGAAACCCGCTAGCAA-3’ |
| Ebre_g052890-3R | 5’-TGACATAACTAATTACATGATCAAACTCTACGTATATTTAAATGA-3’ |
| Ebre_g061651-5F | 5’-TTACAACAAATATAAAAACAATGGAAACAACTACTGTTTATGGTG-3’ |
| Ebre_g061651-3R | 5’-TGACATAACTAATTACATGACTACCAACGCTTAAACTCGTTTG-3’ |
| Ebre_g061652-5F | 5’-TTACAACAAATATAAAAACAATGATACGAGATGTGTTGGCT-3’ |
| Ebre_g061652-3R | 5’-TGACATAACTAATTACATGACTAAAGTTTCTGTAGAATCAAGTG-3’ |
| Ebre_g002073-5F | 5’-TTACAACAAATATAAAAACAATGGATGAGGTAAGAAACACTGT-3’ |
| Ebre_g002073-3R | 5’-TGACATAACTAATTACATGATTAGAGTTTATGCAAAATCAGATTC-3’ |
| Ebre_g038429-5F | 5’-TTACAACAAATATAAAAACAATGTTTAGACAATCAAGAACAAAGC-3’ |
| Ebre_g038429-3R | 5’-TGACATAACTAATTACATGATTAGAGTTTATGCAAAATCAGATTCG-3’ |
| Ebre_g026712-5F | 5’-TTACAACAAATATAAAAACAATGGATGAGTTATATTTCTCTATTTC-3’ |
| Ebre_g026712-3R | 5’-TGACATAACTAATTACATGATCAAGTGCTTGTTGTCAAGAATC-3’ |
| Ebre_g043846-5F | 5’-TTACAACAAATATAAAAACAATGGATCCTTTGTTTATCACTATCG-3’ |
| Ebre_g043846-3R | 5’-TGACATAACTAATTACATGATCAAGCCTGAGATATTAGCTTTTG-3’ |
| Ebre_g005573-5F | 5’-TTACAACAAATATAAAAACAATGCTACCGGGCAAGATATT-3’ |
| Ebre_g005573-3R | 5’-TGACATAACTAATTACATGATCATGTTTGAGACAATAGATTCCC-3’ |
| Ebre_g028301-5F | 5’-TTACAACAAATATAAAAACAATGGAACTCCAAATCCACTTTTCT-3’ |
| Ebre_g028301-3R | 5’-TGACATAACTAATTACATGATTAATTTGTGTTACAAGCAATTGGAAC-3’ |
| Ebre_g047827-5F | 5’-TTACAACAAATATAAAAACAATGGATTATCCAAGCTTCATGTTG-3’ |
| Ebre_g047827-3R | 5’-TGACATAACTAATTACATGATCAAAGTTGGATCGGAACGA-3’ |
| Ebre_g050362-5F | 5’-TTACAACAAATATAAAAACAATGGATTATCCAAGCTTCATATTG-3’ |
| Ebre_g050362-3R | 5’-TGACATAACTAATTACATGATCAAAGTTTGATGGGAACAACC-3’ |
| Ebre_g028780-5F | 5’-TTACAACAAATATAAAAACAATGTTTCTCTCATTTGAGCTCATTC-3’ |
| Ebre_g028780-3R | 5’-TGACATAACTAATTACATGATCATGGAAATATATATTTCAAACCAC-3’ |
| Ebre_g041463-5F | 5’-TTACAACAAATATAAAAACAATGGCTCAAATCTACGGTCC-3’ |
| Ebre_g041463-3R | 5’-TGACATAACTAATTACATGACTAATTGTAAAGAGTAGGTACAAC-3’ |
| Ebre_g049752-5F | 5’-TTACAACAAATATAAAAACAATGGAGATCTTCACTATCTTCCCT-3’ |
| Ebre_g049752-3R | 5’-TGACATAACTAATTACATGATTATATACTAGTCATCTTCTCATTG-3’ |
| Ebre_g035430-5F | 5’-TTACAACAAATATAAAAACAATGTTCCTCGTTGCAAGTCT-3’ |
| Ebre_g035430-3R | 5’-TGACATAACTAATTACATGATCAAATTCGAGTGTAAGGTGTG-3’ |

**Supplementary Table 12 | Primers used in gene knock-out and** **integration**

| **Primer Name** | **Primer Sequence (5’→3’)** |
| --- | --- |
| CIT2-1-5F | 5’-GACCAATGTTAATGAAAACTTGAAC3-3’ |
| CIT2-1-3R (Ura3) | 5’-AATGATGAATTGAATTGAAATTTTCTTGTTACTAGTATTA-3’ |
| CIT2-2-5F (Ura3) | 5’-TAATACTAGTAACAAGAAAATTTCAATTCAATTCATCATT-3’ |
| CIT2-2-3R (Ura3) | 5’-AAAAGTAGGATGTAATCCAACTGATATAATTAAATTGAAGCT-3’ |
| CIT2-3-5F (Ura3) | 5’-AGCTTCAATTTAATTATATCAGTTGGATTACATCCTACTTTT-3’ |
| CIT2-3-3R | 5’-TACTTTCTTGACCACCTCCGTAACT-3’ |
| CIT2-1-3R | 5’-AAAAGTAGGATGTAATCCAATTTTCTTGTTACTAGTATTA-3’ |
| CIT2-2-5F | 5’-TAATACTAGTAACAAGAAAATTGGATTACATCCTACTTTT-3’ |
| MLS1-1-5F | 5’-AATCTTTAGGGAGGGTAAAGTTGGA-3’ |
| MLS1-1-3R (Ura3) | 5’-AATGATGAATTGAATTGAAATTTCTTAATTCTTTTATGTG-3’ |
| MLS1-2-5F (Ura3) | 5’-CACATAAAAGAATTAAGAAATTTCAATTCAATTCATCATT-3’ |
| MLS1-2-3R (Ura3) | 5’-GTACACTGGGGCAAGGGAGACTGATATAATTAAATTGAAG-3’ |
| MLS1-3-5F (Ura3) | 5’-CTTCAATTTAATTATATCAGTCTCCCTTGCCCCAGTGTAC-3’ |
| MLS1-3-3R | 5’-TTTCGGTCCCATTTGGCTGGTTTGC-3’ |
| MLS1-1-3R | 5’-GTACACTGGGGCAAGGGAGATTTCTTAATTCTTTTATGTG-3’ |
| MLS1-2-5F | 5’-CACATAAAAGAATTAAGAAATCTCCCTTGCCCCAGTGTAC-3’ |
| u3-2-5F | 5’-TTTCAATTCAATTCATCATT-3’ |
| u3-2-3R | 5’-CTGATATAATTAAATTGAAGCT-3’ |
| L1-F | 5’ -GTTCTATACTTCTCTCTGCTATACCTACAAGC-3’ |
| L1-R | 5’ -AGGCAATTTTAGAGGGGACTTCTGACTCC-3’ |
| L2-F | 5’ -GAACTGTAATATATAGCTACGCCCTATCTGG-3’ |
| L2-R | 5’ -CCCGAGCTTCAAGCCGATTTAACCGTTG-3’ |
| L3-F | 5’ -TAGACGCCAACTACGCTGAC-3’ |
| L3-R | 5’ -GACTTAGTCCGTTTCTCGGCTATCG-3’ |
| L4-F | 5’ -CCAGACGATACAGAGGCTAAGAATA-3’ |
| L4-R | 5’ -AGGTTCCAACTGCTCTTACTGTAGT-3’ |
| L5-F | 5’ -CAGCCAACCAGTCAGATTAGC-3’ |
| L5-R | 5’ -CGACGAACGAGATACGATAGAAC-3’ |
| L6-F | 5’ -ATTGGACGAGTTCTACCTGACAGAAC-3’ |
| L6-R | 5’ -TTCCGATACGCCTTGTCTCCA-3’ |
| 15-site1-Leu2-F | 5’ -GCCAGGCGCCTTTATATCATATAATTAAGACA-3’ |
| 15-site2-R | 5’ -ATAAAGCAGCCGCTACCAAACAGAC-3’ |
| 17-site1-His3-F | 5’ -GTCGGTCGACTGTGCACAAAGGCCA-3’ |
| 17-site2-R | 5’ -AAAGCTGGCTCCCCTTAGACAAATACGCTA-3’ |
| M13F | 5’ -GTAAAACGACGGCCAGT-3’ |
| M13R | 5’ -CAGGAAACAGCTATGAC-3’ |
| Co-F1 | 5’ -AGGCAAGATAAACGAAGGCAAAG-3’ |
| Co-R1 | 5’-GACACTGTTAGAATGAAGTTCCCAAA-3’ |
| Co-F2 | 5’- TGCTACTAAGGCTATCAAGGAATGG - 3 ’ |
| Co-R2 | 5’- TGGTGTTTGGAAGGCTCACG- 3 ’ |
| Co-F3 | 5’- AACTGTTCTCAAATCTTGGGTTCTACT- 3 ’ |
| Co-R3 | 5’- CATCTTGGACGGTTCTGACTACG- 3 ’ |
| Co-F4 | 5’- AATACAACTACGGTGACTTCATCCC- 3 ’ |
| Co-R4 | 5’- GGCTCCCCTTAGACAAATACG- 3 ’ |
| Co-F5 | 5’- ATCCGAACAACAGAGCATAGG- 3 ’ |
| Co-R5 | 5’- AAGCAAGCTATCGCTATGC- 3 ’ |
| Co-F6 | 5’- CAAGGTGCTATCACTAACCGT- 3 ’ |
| Co-R6 | 5’- GCTTTTTGAGTTTCTGGAATAG- 3 ’ |
| Co-F7 | 5’- ATGTCTATTCCAGAAACTCAAAAAGC- 3 ’ |
| Co-R7 | 5’- ATAAAGCAGCCGCTACCAAACAGAC- 3 ’ |

**Supplementary Note 1**

*Erigeron breviscapus* is a traditional Chinese medicine, which has been used to promote blood circulation and remove blood stasis for people in the Miao nationality in Yunnan province for more than one thousand years. The first record for *E. breviscapus* as plant medicine was done by the socialite Mao Lan in the book “*Austroyunnanese Materia Medica*” during the early stage of Ming Dynasty. Until last century, modern technologies had been used to investigate the pharmacology of *E. breviscapus*. The total flavonoids extract from *E. breviscapus*, has been used to treat cardiovascular and cerebrovascular diseases in clinic since 1970s. With the speeding up of the population aging, cardiovascular and cerebrovascular diseases have become the first death factor in China even in the world. Breviscapine was one of most important cerebrovascular proprietary Chinese medicines. In 2016, approximately 72 million breviscapine injections were produced in China. Considering one patient need seven breviscapine injections for one period treatment, we inferred that the products of breviscapine injections benefit more than 10 million patients each year in China.

**Supplementary References**

1. Ostrowski, M. & Jakubowska, A. Udp-Glycosyltransferases of Plant Hormones. *Advances in Cell Biology* **4**, 43-60 (2014).

2. Kumar, S., Stecher, G. & Tamura, K. MEGA7: Molecular Evolutionary Genetics Analysis Version 7.0 for Bigger Datasets. *Molecular Biology & Evolution* **33**, 1870 (2016).

3. Noguchi, A. et al. Local differentiation of sugar donor specificity of flavonoid glycosyltransferase in Lamiales. *Plant Cell* **21**, 1556-1572 (2009).

4. Ono, E., Ruike, M., Iwashita, T., Nomoto, K. & Fukui, Y. Co-pigmentation and flavonoid glycosyltransferases in blue Veronica persica flowers. *Phytochemistry* **71**, 726-735 (2010).

5. Li, S., Ding, W., Zhang, X., Jiang, H. & Bi, C. Development of a modularized two-step (M2S) chromosome integration technique for integration of multiple transcription units in Saccharomyces cerevisiae. *Biotechnology for Biofuels* **9** (2016).

6. Gietz, R.D. & Akio, S. New yeast- Escherichia coli shuttle vectors constructed with in vitro mutagenized yeast genes lacking six-base pair restriction sites. *Gene* **74**, 527 (1988).

7. Thomas, B.J. & Rothstein, R. Elevated recombination rates in transcriptionally active DNA. *Cell* **56**, 619 (1989).
