# Supplementary material for: Engineering yeast for the production of breviscapine by genomic analysis and synthetic biology approaches
Source: Nat Commun. 2018 Jan 31;9:448. doi: 10.1038/s41467-018-02883-z (PMC5792594; doi:10.1038/s41467-018-02883-z)
Supplement: Supplementary file 3 — Descriptions of Additional Supplementary Files [file 41467_2018_2883_MOESM3_ESM.pdf]

Descriptions of Additional Supplementary Files

File Name: Supplementary Data 1
Description: 312 P450 genes in E. breviscapus genome

File Name: Supplementary Data 2
Description: Protein-coding genes annotation in E. breviscapus

File Name: Supplementary Data 3
Description: DNA sequences used in the study

File Name: Supplementary Data 4
Description: The protein sequences of UGT88 and P450 CYP706 families
